# Pharmacokinetic non-interaction analysis in a fixed-dose formulation in combination of atorvastatin and ezetimibe

**DOI:** 10.3389/fphar.2014.00261

**Published:** 2014-11-27

**Authors:** Omar Patiño-Rodríguez, Irma Torres-Roque, Maricela Martínez-Delgado, Abraham Escobedo-Moratilla, José Pérez-Urizar

**Affiliations:** ^1^Dixpertia, Investigación Biofarmacéutica y Farmacológica S.C., San Luis PotosíMéxico; ^2^Laboratorio de Farmacología y Fisiología, Facultad de Ciencias Químicas, Universidad Autónoma de San Luis PotosíSan Luis Potosí, México

**Keywords:** pharmacokinetic drug–drug interaction, atorvastatin, ezetimibe, statins, LC-MS-MS

## Abstract

Recent clinical research has shown that atorvastatin (ATO) in combination with cholesterol absorption inhibitor ezetimibe (EZE) significantly reduces LDL-C level in patients with hypercholesterolemia, showing a superior lipid-lowering efficacy compared to statin alone. With no information currently available on the interaction between the two drugs, a pharmacokinetic study was conducted to investigate the influence of EZE on ATO and conversely when the two drugs were coadministered. The purpose of this study was to investigate the presence of differences in the pharmacokinetic profiles of capsules containing ATO 80 mg, EZE 10 mg or the combination of both 80/10 mg administered to healthy Mexican volunteers. This was a randomized, three-period, six-sequences crossover study. 36 eligible subjects aged between 20 to 50 years were included. Blood samples were collected up to 96 h after dosing, and pharmacokinetic parameters were obtained by non-compartmental analysis. Adverse events were evaluated based on subject interviews and physical examinations. Area under the concentration-time curve (AUC) and maximum plasma drug concentration (C_max_) were measured for each drug alone or together and tested for bioequivalence-based hypothesis. The estimation computed (90% confidence intervals) for AUC and C_max_, were 96.04% (85.88–107.42%) and 97.04% (82.36–114.35%), respectively for ATO–EZE combination versus ATO alone, while 84.42% (77.19–92.32%) and 95.60% (82.43–110.88%), respectively, for ATO–EZE combination versus EZE alone were estimated. These results suggest that ATO and EZE have no relevant pharmacokinetic drug–drug interaction.

## INTRODUCTION

Atorvastatin (ATO) is a member of a lipid-lowering family of agents called statins, is a synthetic reversible inhibitor of 3-hydroxy-3-methylglutaryl-coenzyme A (HMG-CoA) reductase; the rate-limiting enzyme in cholesterol biosynthesis (Liu et al., 2010). This HMG-CoA reductase inhibitor can efficiently and dose dependently lower both cholesterol (25–61%) and triglyceride (9–61%) levels in hyperlipidemic patients, (Nawrocki et al., 1995; Heinonen et al., 1996) and produces a significantly larger reduction (38–51%; *P* ≤ 0.01) of colesterol and triglycerides compared with the equivalent doses of other statin drugs (Jones et al., 1998). Following oral administration, ATO is rapidly absorbed, and maximum plasma concentrations are achieved within 1–2 h. ATO is extensively metabolized by cytochrome P450 3A4 to active metabolites: ortho- and parahydroxy ATO. Approximately 70% of the circulating inhibitory activity for HMG-CoA reductase is attributed to these active metabolites (Pfizer Inc. Lipitor^®^, 2009). Although the pharmacokinetics or bioavailability of ATO has been previously studied in other populations (Posvar et al., 1996; Koytchev et al., 2004; Bahrami et al., 2005; Liu et al., 2010), a few studies were reported in Mexican subjects.

Ezetimibe (EZE) is the first of the cholesterol absorption inhibitors, a novel class of lipid modifying drugs, which potently inhibit the absorption of biliary and dietary cholesterol from the small intestine without affecting the absorption of fat-soluble vitamins, triglycerides or bile acids (van Heek et al., 2000). Following oral administration EZE is readily absorbed and glucuronidated (EZE-G) in the intestinal epithelium, that is pharmacologically active phenolic glucuronide. EZE-G enters an enterohepatic recirculation reaching dual peak blood levels at 4–6 and 10–12 h, suggesting enterohepatic recirculation of EZE and conjugated form (van Heek et al., 2000; Patrick et al., 2002; Malloy and Kane, 2007; Mahley and Bersot, 2008). The drug and its metabolite are eliminated slowly; with terminal elimination half-life of 20–30 h (Ezzet et al., 2001). EZE is used as monotherapy or in combination with statins for the reduction of elevated levels of TC, LDL-C, and Apo B in patients with primary hypercholesterolaemia (Mahley and Bersot, 2008). EZE had no effect on the activity of major drug metabolizing enzymes (CYP450), which reduces any potential drug–drug interactions with other medications (van Heek and Davis, 2002).

The possibility of combining cholesterol-lowering medications to attain greater LDL-C lowering is therefore an important therapeutic option for more effective intervention on cardiovascular risk reduction for high–and very high–risk patients. One currently available approach is the simultaneous intervention by two complementary mechanisms regulating plasma colesterol levels: intestinal cholesterol absorption and hepatic cholesterol synthesis (Ballantyne et al., 2005). The combined use of ATO–EZE and the study of non-interaction between the two formulations is an interesting alternative for the treatment of patients with hypercholesterolemia and cardiovascular disease risk.

On the other hand, several analytical methods exist for analysis of ATO (Bahrami et al., 2005; Bhatt et al., 2010), and EZE (Sistla et al., 2005; Li et al., 2006; Bahrami et al., 2010). In accordance to Draft Guidance published for Food and Drug Administration’s (FDA’s), the Schuirmann hypothesis is based in ATO in human plasma, and the ATO metabolites only provide complementary information about metabolism of drug in the organism (Rowland and Tozer, 2011). For the EZE the Draft Guidance (FDA’s) considers only the total EZE quantified in human plasma.

Therefore, the aim of the present study was to investigate the presence of differences in the pharmacokinetics profiles of a fixed-dose formulation alone and in combination of ATO (80 mg) and EZE (10 mg) in oral dose in healthy fasted Mexican volunteers, using a bioequivalence-based hypothesis to perform a non-interaction analysis between the two assessed drugs.

## MATERIALS AND METHODS

### STUDY DESIGN AND PROCEDURES

The study was a randomized, open-label, crossover type Williams (1949), prospective longitudinal a single dosage not therapeutic of 80 mg of ATO (Treatment A), 10 mg of EZE (Treatment B) or a combination of both (Treatment AB), trial in healthy Mexican subjects under fasting condition. Thirty six healthy Mexicans volunteers of both gender who were between the ages of 18 and 45 (mean ± SEM: 24.71 ± 0.03 years), had heights between 140.0 and 190.0 cm (163.0 ± 0.005 cm), and weighed between 43.50 and 79.50 kg (62.15 ± 1.9 kg) were enrolled in the study. The study protocol was approved by an independent ethics committee as well as by the regulatory authority in Mexico (COFEPRIS), and it was conducted following the ethical principles described in the Declaration of Helsinki.

Subjects received formulations in three separate sessions according to the scheme shown in the **Table 1**, with 14 day washout between sessions. The demographic characteristics of the volunteers are presented in **Table 1**.

**Table 1:** Demographic characteristics and formulation sequence of administration of the healthy Mexican volunteers.

Volunteer	Sex	Age (y)	Height (cm)	Weight (kg)	BMI (%)	Sequence (period)		
						I	II	III
1	Male	24	162	62	23	AB	B	A
2	Female	29	156	55	22	AB	B	A
3	Male	18	168	60	21	A	AB	B
4	Male	24	182	78	23	AB	A	B
5	Female	26	170	60	20	AB	A	B
6	Male	44	171	67	22	A	B	AB
7	Female	37	162	70	26	A	AB	B
8	Male	34	164	67	24	AB	B	A
9	Male	24	172	66	22	A	AB	B
10	Male	43	173	71	23	A	B	AB
11	Female	33	172	63	21	A	B	AB
12	Male	23	166	56	20	B	AB	A
13	Male	32	178	83	26	A	AB	B
14	Female	25	156	50	20	B	A	AB
15	Male	24	167	62	22	AB	A	B
16	Male	37	165	73	26	AB	B	A
17	Male	24	171	74	25	B	A	AB
18	Female	34	155	62	25	A	B	AB
19	Male	27	183	78	23	AB	A	B
20	Male	25	167	71	25	B	AB	A
21	Male	26	166	60	21	B	A	AB
22	Female	20	166	59	21	A	AB	B
23	Female	19	168	60	21	A	AB	B
24	Male	32	159	67	26	B	A	AB
25	Male	49	150	53	23	A	B	AB
26	Male	26	172	75	25	AB	B	A
27	Male	21	185	76	22	B	AB	A
28	Female	32	168	68	24	AB	B	A
29	Male	18	183	67	20	B	AB	A
30	Male	27	165	61	22	B	A	AB
31	Male	46	167	65	23	A	B	AB
32	Male	21	169	67	23	B	AB	A
33	Female	29	143	45	22	B	AB	A
34	Male	30	172	67	22	B	A	AB
35	Female	21	162	57	21	AB	A	B
36	Female	25	150	55	24	AB	A	B

*Tablets containing 80 mg Atorvastatin (A), Ezetimibe 10 mg (B), Atorvastatin/Ezetimibe 80/10 mg respectively (AB). BMI, body mass index.*

### BLOOD SAMPLING

Blood samples (4 mL) were collected from a suitable forearm vein by an indwelling catheter or by immediate venipuncture at the following time points: 0.0 (before administration), 0.25, 0.50, 0.75, 1.0, 1.5, 2.0, 2.25, 2.50, 3.0, 3.5, 4.0, 5.0, 6.0, 8.0, 12.0, 24.0, 48.0, 72.0, and 96.0 h after study drug administration. Prior to each sample collection, 1 mL of blood was drawn and discarded. After sampling, the catheter was flushed with 0.8 mL of sodium heparin (25 IU/mL) to ensure patency. Blood samples were drawn into pre-labeled heparin containing tubes and plasma samples were separated within 30 min after drawing by centrifugation at 3000 rpm for 10 min at room temperature. Plasma was stored frozen (≤–20°C) in labeled polypropylene tubes until analysis.

### MATERIALS AND REAGENTS

Analytical standards of calcium ATO and IS (paroxetine), EZE and IS (hydrochlorothiazide) were kindly donated by a pharmaceutical company (Laboratorios SENOSIAIN, S. A. de C. V.). β-glucuronidase from *Helix pomatia* (Sigma-Aldrich, USA), acetonitrile MS grade (EMD Chemicals, USA) and ammonium formiate HPLC grade (Fluka, USA) were acquired with local distributors. All working solutions in this study were prepared with deionized water.

### DRUG FORMULATIONS

The test and reference formulation was manufactured by Laboratorios SENOSIAIN, S. A. de C. V. (Mexico). A formulation of ATO capsules (40 mg), EZE capsules (10 mg), and ATO–EZE capsules (40 mg/10 mg) were used in the pharmacokinetic study available in batches with valid certificates of analysis and were kept in a sealed container at a controlled room temperature of 15–25°C until further use.

### QUANTIFICATION OF PLASMA CONCENTRATIONS OF ATO AND TOTAL EZE BY LC-MS/MS METHOD

For the determination of both molecules were developed an analytical method, where only once plasma processing is used to obtain both molecules, but the quantification of the analytes was performed on two separate injections ATO in positive electrospray ionization ESI (+) mode and EZE in negative electrospray ionization ESI (-) mode. The calibration curve used to determination of ATO was in the range 1–60 ng/mL, the paroxetine (10 μg/mL) was used as internal standard, the range used to EZE is 0.5–100 ng/mL, and the hydrochlorothiazide (10 μg/mL) was used as internal standard.

### SAMPLE PREPARATION

Frozen plasma samples were thawed at room temperature. A 0.3 mL aliquot of human plasma was spiked with each stock solution (5 μL of ATO and 5 μL of EZE) of calibration curve samples and quality control samples, and IS (5 μL of paroxetine and 5 μL of hydrochlorothiazide) solution. Then, 0.5 mL sodium acetate buffer 0.025 M pH 5.0 was added. Ten microliters of β-glucuronidase from *Helix pomatia* (>90000 UI/mL) was added, the mixture was vortexed for 0.5 min. The mixture was incubated at 40 ± 3°C from 1 h. After incubation the reaction was finished with 100 μL of NaOH 0.1N. One mL of ethyl acetate was added to extract analytes, the mixture was vortexed for 1.0 min. After mixing, the samples were frozen for 5 min at -80°C, after centrifuged for 5 min at 14,000 rpm with a bench-top centrifugal separator (Eppendorf 5418, Germany). A total volume of the organic extract was evaporated to dryness under a stream of nitrogen and reconstituted in 300 μL of acetonitrile:water (50:50). The total volume was transferred to a glass autosampler vial and a 2.0 μL aliquot of the solution was injected into the LC-MS/MS system for analysis.

### LC-MS/MS AND CONDITIONS

Chromatographic analysis was performed on UPLC-MS/MS system consisting of Acquity UPLC coupled to a tandem mass spectrometry detector XEVO-TQS (Waters, USA) and Acquity UPLC BEH C18 (1.7 μm, 2.1 × 100 mm) column (Waters, USA). The mobile phase consisted of an acetonitrile-5 mM ammonium formiate buffer solution (80:20, v/v) at 0.2 mL/min flow rate. The run time was 2.6 min; the sample volume injected was 2.0 μL. The column temperature was set to 40°C. The autosampler cooler was set at 8°C. For ATO the mass spectrometer was set in multiple reactions monitoring (MRM) mode in ESI positive ionization mode. Collision energy and cone voltage were 12 and 19 V, respectively. Cone and desolvation gas flow rate were set to 150 and 600 L/min, respectively, using Argon as collision gas at flow rate of 0.15 mL/min. Tandem mass spectrometer was tuned to monitor m/z 559.25 → m/z 440.30 transition for ATO and m/z 330.10 → m/z 192.20 transition for the IS (paroxetine), with dwell time of 0.3 s. For EZE the mass spectrometer was set in MRM mode in ESI negative ionization mode. Collision energy and cone voltage were 12 and 15 V, respectively. Tandem mass spectrometer was tuned to monitor m/z 408.15 → m/z 271.20 transition for EZE and m/z 296.0 → m/z 269.00 transition for the IS (hydrochlorothiazide), with dwell time of 0.2 s. MRM data were acquired and analyzed through MassLynx software (Waters, USA).

### ASSAY VALIDATION

The analytical method was validated according to criteria established by the Mexican Regulatory Guidelines (NOM-177-SSA1-1998, 2013). Drug-free plasma was spiked with ATO and EZE solution to obtain a calibration curve. In the same manner, QC samples (points) were prepared at low, medium, and high concentration levels (6.0, 24.0, and 52 ng/mL for ATO and 5.0, 50.0, and 90 ng/mL for EZE), and these were employed to determine absolute recovery and intra- and interday precision and accuracy. Selectivity was evaluated by preparing the lower limit of quantitation (LLOQ) in lipemic or hemolyzed plasma and by spiking drug-free plasma with ciprofloxacin, paracetamol, difenidol, ranitidine, and caffeine. Stability [biological matrix at –70°C, bench-top at room temperature (20°C), three freeze-and-thaw cycles, enzymatic reaction at 40°C for 1 h, and processed samples inside the autosampler] was also evaluated.

### PHARMACOKINETIC ANALYSIS

Pharmacokinetic parameters for ATO and EZE were calculated using non-compartmental and compartmental models with WinNonlin 6.2.1 software (Pharsight, Mountain View, CA, USA, 2011). From the individual data, it was estimated the pharmacokinetic parameters of ATO and EZE. The Maximum plasma concentration (C_max_), time to reach C_max_ (T_max_), area under the plasma concentration-time curve (AUC) from time 0 to the time of the last measurable concentration (AUC_0-t_) and AUC extrapolation to infinity (AUC_0-∞_) was calculated according to the non-compartmental method. For estimation of the absorption rate constant (Ka), half-life of the absorption process (T_1/2_ abs) as well as the disposition and elimination parameters: apparent volume of distribution (V/F), clearance apparent (CL/F), elimination rate constant (Ke), and elimination half-life (T_1/2_), the best model that described the individual pharmacokinetic data was fitted as an open model of one compartment with first order absorption without lag-time.

### STATISTICAL ANALYSIS

In accordance with the Mexican Regulatory Guidelines (NOM-177-SSA1-1998, 2013), 36 volunteers were the minimum sample required (assuming an 80% power to detect a 20% difference). An ANOVA for a 3 × 6 crossover design was performed on the decimal logarithm-transformed parameters C_max_, AUC_0-t_, and AUC_0-∞_ to evaluate fixed effects such as period, sequence, formulation, and carryover. Logarithm-transformed values of these parameters were considered to construct a classic CI at 90%, with *P* < 0.05 indicating significance. The formulations were considered bioequivalent if the 90% CI of the logarithm-transformed ratios (test/reference) of C_max_ (an index of the rate of absorption), AUC_0-t_, and AUC_0-∞_ (indexes of extent of absorption) were within the predefined range of 0.80–1.25.

## RESULTS

### ANALYTICAL METHOD

We measure the total concentration of EZE, after incubation of plasma samples in the presence of β-glucuronidase enzyme. Thus, after enzymatic treatment of the plasma levels of total EZE and ATO were quantified with a method of Ultraperformance Liquid Chromatography coupled to mass spectrometry, using an analytical method developed and validated, through a technique of liquid-liquid extraction. The biological samples were analyzed by liquid chromatography with separate injections for each analyte (ATO and EZE) and validated according to the Mexican Guideline (NOM-177-SSA1-1998, 2013). The method was selective, robust and satisfies the stability requirements evaluated during validation.

### PHARMACOKINETIC PROFILES

The data was analyzed under the null hypothesis of interaction (bioequivalence-based hypothesis), we expect the average time courses by period suggest an increase or reduction of significant magnitude at the levels reached by the drug when administered in combination compared to when administered alone. The potential pharmacokinetic interactions should be explained not only in terms of magnitude, also with the factor that explains the difference to propose the type and magnitude of the implication that such interaction would have on the effectiveness and drugs safety (Hauschke et al., 2007; Zhang et al., 2009).

**FIGURE 1:**
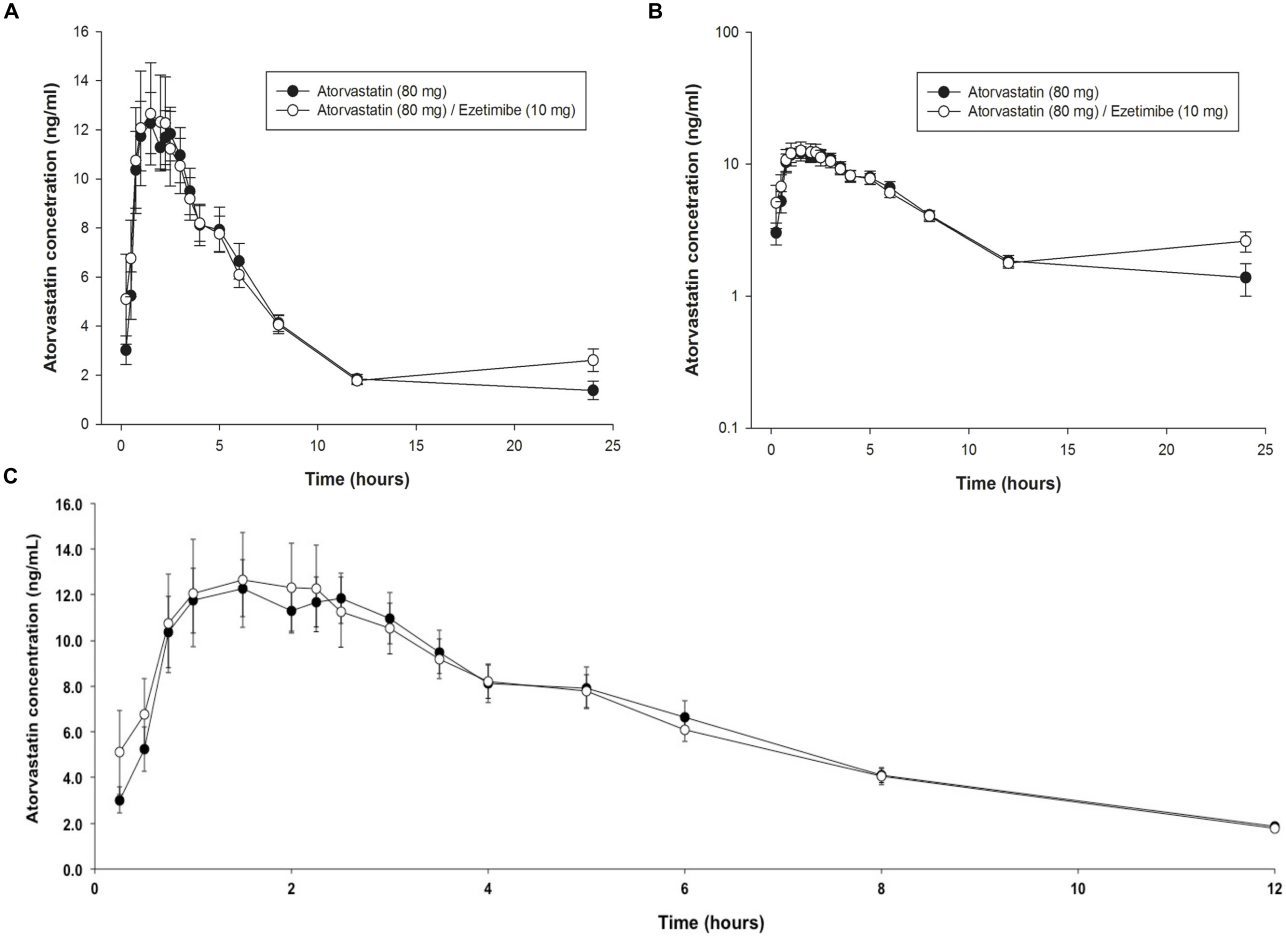
**Time-course of plasma levels of atorvastatin (80 mg) when given alone or in combination with 10 mg of ezetimibe in healthy subjects. (A)** Arithmetic profile 025 h; **(B)** Semilogarithmic profile 025 h; **(C)** Arithmetic profile 012 h.

**FIGURE 2:**
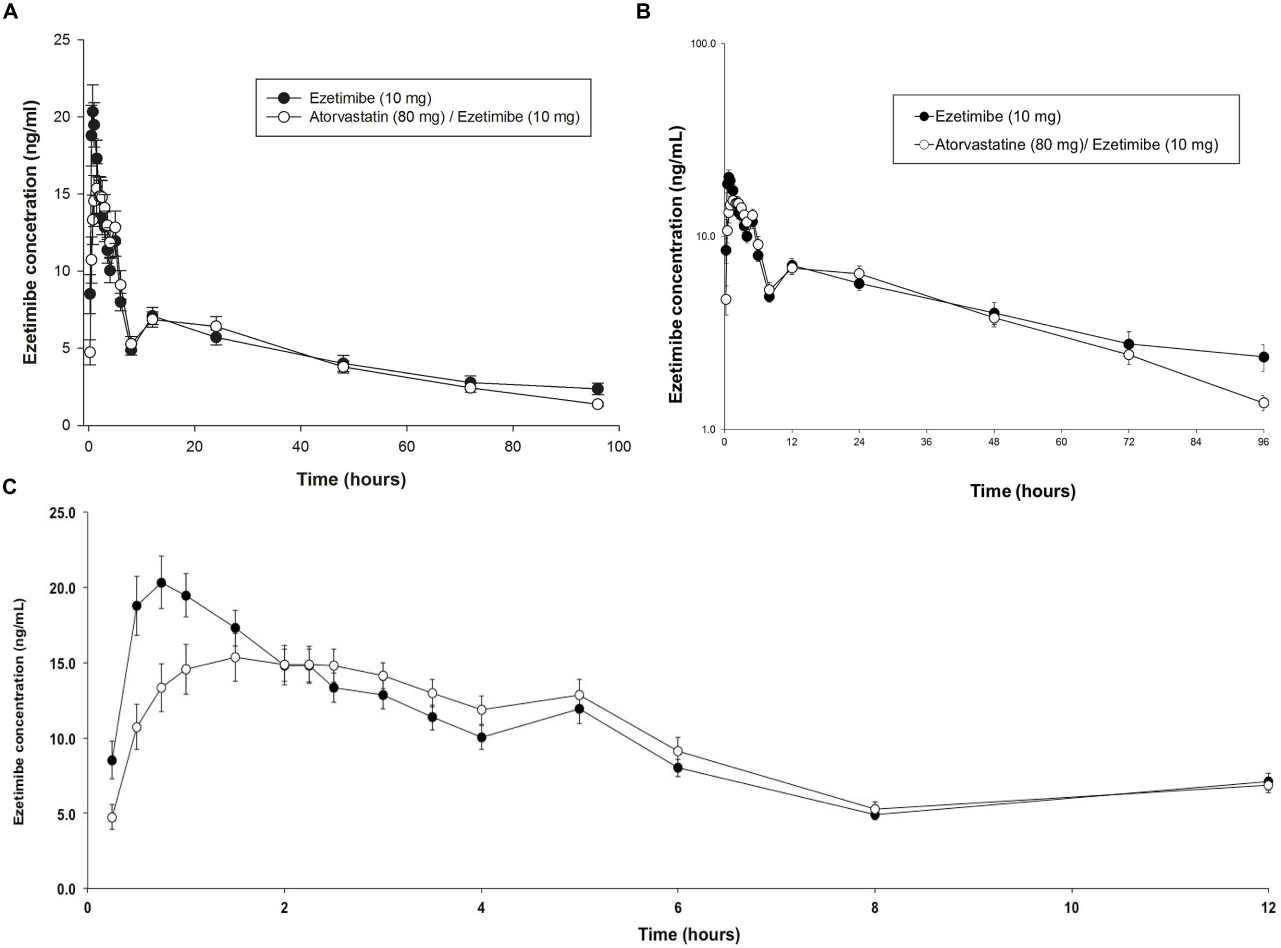
**Time-course of plasma levels of ezetimibe (10 mg) when given alone or in combination with 80 mg of atorvastatin in healthy subjects. (A)** Arithmetic profile 0–96 h; **(B)** Semilogarithmic profile 0–96 h; **(C)** Arithmetic profile 0–12 h.

In **Figures 1A and 2A** the average time courses in arithmetic scale, the plasma concentration of ATO and EZE are comparatively shown in each of the periods in which volunteers received the drug reference (ATO or EZE alone) or test drug (ATO–EZE). From **Figure 1A** shows that on average, the time profiles of ATO plasma concentrations were similar between the reference formulation and test. With both formulations C_max_ values reached 11–13 ng/mL which reached around 1.5–2.5 h, which is better appreciated in **Figure 1C** (0–12 h). When the course ATO pharmacokinetic profile was analyzed on a logarithmic scale (**Figure 1B**) were found that curves generated for both products (test and reference) overlapped.

For EZE, in **Figures 2A,B** the time courses of the plasma in both logarithmic and arithmetic scale as shown. In both cases, it can be seen that the average peak concentration EZE is higher and reaches a little faster when given alone compared to when given in combination with ATO (**Figure 2C**). However, from the second hour no differences in drug levels between formulations, including the double peak that appears between the 4 and 6 h, corresponding to enterohepatic recirculation of EZE (Kosoglou et al., 2005) which is very similar between test and reference products.

### PHARMACOKINETIC PARAMETERS

Through the non-compartmental estimation of pharmacokinetic parameters (**Table 2**) showed that the average maximum concentration (±SD) of ATO in formulating Test (combination ATO–EZE) is slightly higher (19.80 ± 14.52 ng/mL) that produced only when the drug is given: ATO (18.78 ± 8.89 ng/mL), but is achieved at a similar time (T_max_: 2.03 ± 1.31 h) compared to that observed with the reference formulation (T_max_: 2.08 ± 1.41 h). The above results are in agreement with the reported for Lennernäs (2003), which states that after doses of 20 and 40 mg of ATO C_max_ of 6.9 is reached at 12.7 ng/mL 1–2 h. In the case of values of AUC_0-t_ 76.79 ± 44.47 ng × h/mL and 76.01 ± 36.09 ng × h/mL; while the AUC_0-inf_ were 89.08 ± 48.97 ng × h/mL and 87.42 ± 40.47 ng × h/mL for the test formulations (ATO–EZE combination) and reference (ATO) respectively. In this regard, results of other authors describes that after 80 mg dose AUC_0-t_ reached is of the order of 102–134 ng × h/mL (Jacobson, 2004).

**Table 2:** Statistical evaluation of non-interaction for Atorvastatin bioavailability parameters in volunteers who received administration of the formulations evaluated: A: Atorvastatin 80 mg; AB [fixed dose combination (80 mg Atorvastatin/Ezetimibe 10 mg)].

	A vs. AB (*n* = 36)						
	Formulation A reference	Formulation B test	Ratio B/A %	Westlake interval 90%	Clasic interval 90%	Unilateral double test Schuirmann (*p* < 80; *p* > 120 *p* total)		
Ln C_max_	2.82	2.79	97.04	83.53	82.36	0.0273	0.0067
	0.09	0.09		116.47	114.35	0.0340	
Ln AUC_ 0-t_	4.23	4.19	96.04	87.66	85.88	0.0046	0.0002
	0.08	0.08		112.34	107.42	0.0048	
Ln AUC_0-inf_	4.37	4.36	98.21	88.81	87.91	0.0018	0.0004
	0.08	0.08		111.19	109.71	0.0022	

*C_max_, Maximum concentration; AUC_0-t_, area under curve to the final observation; AUC_0-inf_, area under curve extrapolated to infinity.*

In order to compare the process of elimination of ATO after administration of both products, the following values for the elimination half-life were estimated: 4.23 ± 2.17 h for the combination ATO–EZE 3.97 ± 2.03 h and when the drug was administered alone (ATO).

Furthermore, when comparing the individual results, regarding the ATO–EZE combination in this study, it was confirmed that reported by the manufacturer of the first product registered for the combination (Zhu et al., 2001; Kosoglou et al., 2002; Zetia, 2014), there seems to be a clinically relevant pharmacokinetic interaction. Furthermore, in the case of EZE, the C_max_ of the drug in the subjects treated with the combination ATO–EZE (21.09 ± 8.57 ng/mL) were 17.3% lower on average than that produced when the drug is administered alone: EZE (24.76 ± 10.27 ng/mL) and was reached at a longer period [T_max_: 2.33 ± 1.46 h with respect to the reference formulation (EZE) 1.33 ± 1.20 h]. The bioavailability of EZE measured by AUC_0-t_ showed that the combination with 385.83 ± 186.36 ng × h/mL are reached values very similar to the drug alone (386.82 ± 175.37 ng × h/mL). While, AUC_0-inf_ was 432.57 ± 206.49 and 454.25 ± 200.01 ng × h/mL for the test formulations (ATO–EZE combination) and reference (EZE) respectively. In clear correspondence with previous results, bioavailability was observed that the half-life of the terminal elimination phase was 22.88 ± 12.61 h for ATO–EZE and 23.58 ± 12.14 h for the reference product: EZE. These results led to the proposition that there must be some pharmacokinetic interaction in the absorption process for EZE. Kosoglou et al. (2002) reported that the parent drug is rapidly absorbed and is biotransformed into glucuronide active metabolite (>80%), and reaches the maximum levels between 1 and 2 h post-administration, involved a enterohepatic recirculation and slow elimination. However, the levels of EZE shown by the author to the dose of 10 mg are generally higher than those achieved in the present study, in particular mentioned that the C_max_ is 64.2–73.6 ng/mL, which is reached between 1.2 and 2.3 h, while the area under the curve to the end point 440–722 ng × h/mL.

## DISCUSSION

Due to the clinical study design, we use statistical approach based on the Schuirmann hypothesis (bioequivalence-based hypothesis) using pharmacokinetic parameters of drugs. The compliance with this hypothesis demonstrated that no pharmacokinetic drug interaction occurred in the present combination.

In order to establish the approval of the hypothesis (non-interaction), intervals classic symmetrical Westlake (90%) confidence were calculated, and the unilateral double test Schuirmann was applied to the logarithmic transformation of the C_max_ parameters AUC_0-t_, AUC_0-inf_ of ATO and EZE in the evaluated formulations. Corresponding analyzes were performed using the program WinNonlin (WinNonlin 6.2.1, 2011).

The results of **Table 2** show there is no difference between the single administrations of ATO with respect to when given in combination with EZE, means no pharmacokinetic interaction. However, in the case of parameter C_max_ is close to the established acceptance criteria (≥0.8). In this regard, ATO is a molecule has shown an intra-subject variability >30%. The Lipitor drug, shows a high intra-individual variability according to diverse regulatory bodies such as the European Medicines Agency [EMEA] (2010), in this sense, is accurate to extend the acceptance criteria of the confidence interval at 90% for C_max_ parameter values between 0.75 and 1.33. Under this consideration is re-analyzed the data and confirmed that using the expansion of the range for the parameter C_max_ power, in this pharmacokinetic parameter, is >0.8 criterion established regulatory acceptance, which would lead to establish that using this strategy statistical analysis, one can conclude that the presence of EZE does not produce an interaction on the pharmacokinetics of ATO. The chemical nature of ATO and the mechanism of action of EZE are probably the reasons behind this finding, because EZE inhibits the absorption of biliary and dietary cholesterol, but apparently it does not have the ability to inhibit the absorption of ATO, even though the polarity of the molecule of ATO is low (Drugbank, 2014). Furthermore there is not an increase in the bioavailability or a change in the elimination rate, so this suggests that EZE does not have any effect in the pharmacokinetic profile of ATO at the administered doses.

For EZE the **Table 3** shows that for the C_max_ parameter (log-transformed), the ratio test: reference has an average value of 84.42%, means that not exceeded the expectation of ±20%, considered conventional as the observed differences translate into relevant clinically implication (Hauschke et al., 2007). However, the estimation of the confidence interval as both Westlake Classic 90%, as well as double-sided Schuirmann test suggests that the test product does not meet the criteria (80–125%). In contrast, the assessment of the areas under the curve shows that both the ratio test: reference (95.60 and 91.17%) as tests and confidence intervals Schuirmann meet or are close to the level of compliance to consider the approval of the hypothesis. However, it is important to clarify that this is not a bioequivalence study, but analysis consist in a bioequivalence-based hypothesis to analyze the potential pharmacokinetic interaction between the components of the combination ATO–EZE (80/10 mg).

**Table 3:** Statistical evaluation of non-interaction for the parameters of bioavailability of Ezetimibe in volunteers who received administration of the formulations evaluated: B: Ezetimibe 10 mg; AB [fixed dose combination (80 mg Atorvastatin/Ezetimibe 10 mg)].

	B vs. AB (*n* = 36)						
	Formulation A reference	Formulation B test	Ratio B/A%	Westlake interval 90%	Classic interval 90%	Unilateral double test Schuirmann (*p* < 80; *p* > 120 *p* total)		
Ln C_max_	3.14	2.97	84.42	78.78	77.19	0.1585	0.0000
	0.07	0.07		121.22	92.32	0.1585	
Ln AUC_ 0-t_	5.86	5.82	95.60	84.37	82.43	0.0250	0.0022
	0.07	0.07		115.63	110.88	0.0271	
Ln AUC_0-inf_	5.90	5.84	93.91	83.62	81.28	0.0345	0.0010
	0.07	0.07		116.38	108.50	0.0355	

*C_max_, Maximum concentration; AUC_0-t_, area under curve to the final observation; AUC_0-inf_, area under curve extrapolated to infinity.*

Although it is reported that EZE, has not significant interactions in plasma levels in combination with statins (Kosoglou et al., 2002) in our study we found at least an increase in the C_max_ level of EZE (10 mg) when administered with 80 mg of ATO. Nevertheless, this finding could be relevant based on a previous analysis of pharmacodynamic parameters in a population (Kakara et al., 2014) which suggests in a simulation that Rosuvastatin when combined with EZE have a superior clinical response due to a decrease in the LDL synthesis. In fact, the beneficial effects of this combination (ATO–EZE) have been studied at preclinical (Van Rooyen et al., 2013) and clinical level (Bennett et al., 2004; Okada et al., 2012).

On the other hand, the possible mechanism to explain the change in the C_max_ of EZE when administered with ATO is unknown and this study was not designed to answer this situation. However, the difference could be induce by an alternative mechanism of action of ATO that clearly affects the bioavailability and not to the elimination rate, because the AUC’s measures remain with no changes.

## CONCLUSION

No evidence of interaction was found, in the pharmacokinetic process to ATO (80 mg) produced by the co-administration of EZE (10 mg), however, if small differences in C_max_ EZE (10 mg) were documented after the co-administration of 80 mg of ATO, which are not sufficient to conclude that ATO is capable of producing a reduction in the absorption of EZE, especially that the areas under the curve (overall drug exposure) no showing difference according to the strategy statistics employed bioequivalence-based hypothesis (Schuirmann hypothesis).

## Conflict of Interest Statement

The authors declare that the research was conducted in the absence of any commercial or financial relationships that could be construed as a potential conflict of interest.
